# The effects of EPA and DHA enriched fish oil on nutritional and immunological markers of treatment naïve breast cancer patients: a randomized double-blind controlled trial

**DOI:** 10.1186/s12937-017-0295-9

**Published:** 2017-10-23

**Authors:** Elemárcia Martins da Silva Paixão, Ana Carolina de M. Oliveira, Nathalia Pizato, Maria Imaculada Muniz-Junqueira, Kelly G. Magalhães, Eduardo Yoshio Nakano, Marina K. Ito

**Affiliations:** 1 Post Graduate Program in Human Nutrition, University of Brasília, Brasilia, Federal District 70910-900 Brazil; 2 Laboratory of Cellular Immunology, Department of Pathology, Faculty of Medicine, University of Brasilia, Brasilia, Federal District 70910-900 Brazil; 3 Laboratory of Immunology and Inflammation, Department of Cell Biology, Institute of Biology, University of Brasilia, Brasilia, Federal District 70910-900 Brazil; 4 Department of Statistics, University of Brasilia, Brasilia, Federal District 70910-900 Brazil

**Keywords:** Breast cancer, N-3 fatty acids, Fish oil, Immunonutrition, Cytokines, Eicosapentaenoic acid (EPA)

## Abstract

**Background:**

We evaluated the effects of eicosapentaenoic (EPA) and docosahexaenoic (DHA) acids enriched fish oil (FO) on nutritional and immunological parameters of treatment naïve breast cancer patients.

**Methods:**

In a randomized double blind controlled trial, the FO group (FG) patients were supplemented with 2 g/ day of FO concentrate containing 1.8 g of n-3 fatty acids during 30 days. The placebo group (PG) received 2 g/ day of mineral oil. At baseline and after the intervention, plasma levels of n-3 fatty acids, dietary intake, weight, body composition, biochemical and immunological markers were assessed.

**Results:**

At the end of the intervention period, no between group differences were observed regarding anthropometric parameters. There was a significant increase in the plasma phospholipid EPA (*p* = 0.004), DHA (*p* = 0.007) of the FG patients. In FG patients the percentages of peripheral blood CD4^+^ T lymphocytes and serum high sensitivity C-reactive protein (hsCRP) levels were maintained while in PG patients there was a significant increase in hsCRP (*p* = 0.024). We also observed a significant reduction in the percentage of CD4^+^ T lymphocytes in the peripheral blood (*p* = 0.042) of PG patients. No changes in serum proinflammatory cytokine and prostaglandin E_2_ levels were observed.

**Conclusions:**

Supplementation of newly diagnosed breast cancer patients with EPA and DHA led to a significant change in the composition of plasma fatty acids, maintained the level of CD4^+^ T cells and serum levels of hsCRP, suggestive of a beneficial effect on the immune system and less active inflammatory response.

**Trial registration:**

Brazilian Clinical Trials Registry (REBEC): RBR-2b2hqh. Registered 29 April 2013, retrospectively registered.

## Introduction

Cell-mediated immune response (IR) plays an important role in cancer immunoediting. CD4^+^ and CD8^+^ T cells are the main lymphocytes involved in cell-mediated immunity. It is established that an effective anti-tumor IR requires the participation of both types of T lymphocytes cells, since the CD4^+^ T cells are critical for the generation of tumor-specific cytotoxic T cells as well as memory T cells expansion [1]. Fatty acids are modulators of lymphocyte functions. Both the type of fatty acids present in the diet and their serum levels may influence lymphocyte proliferation, cytokine production and T-lymphocyte migration [2, 3].

Breast cancer patients have altered cell-mediated IR compared to healthy controls. In newly diagnosed patients, low peripheral blood CD4^+^ cell counts have been observed [4, 5]. Moreover, results contrary to the above [6] or those who showed no difference between patients and controls [7] have also been published. Furthermore, even in the early stages, breast cancer patients have increased serum levels of prostaglandin E_2_ (PGE_2_) [8] and proinflammatory cytokines such as tumor necrosis factor alpha (TNF-α), interleukin (IL)-1β and IL-6 [9]. Elevated serum levels of C-reactive protein (CRP) at the time of diagnosis were observed and associated with shorter disease-free survival and overall survival of breast cancer patients [10].

Diagnosis of cancer can motivate patients to alter their dietary habits on its own. Nutritional supplement intake such as fish oil, which is the principal source of n-3 fatty acids eicosapentaenoic acid (EPA) and docosahexaenoic acid (DHA), is highly prevalent among breast cancer patients [11]. The benefit of n-3 fatty acid intake on breast cancer incidence has been reported in a recent systematic review of prospective cohort studies, that suggested a dose response relationship of 5% lower risk for each 0.1 g/day increment of marine n-3 fatty acid intake [12]. For those already with the disease, specific human intervention studies are limited and the results have been variable [13–15]. In metastatic breast cancer patients, oral supplementation with DHA during chemotherapy potentially improved patient survival [13] and in patients under chemo or radiotherapy for other types of cancer, the supplementation with EPA and DHA increased body weight [16] and reduced serum CRP [17], proinflammatory cytokines and PGE_2_ levels [14, 16, 17]. However, there is a gap of knowledge on the potential benefit of n-3 fatty acid intake for breast cancer patients at early stages of treatment.

Thus, the aim of this study was to investigate whether supplementation with EPA and DHA, immediately following the diagnosis of breast cancer but prior to treatment, would have a positive impact on patient’s nutritional and selected immune parameters.

## Materials & methods

### Study population

Breast cancer patients attending the University Hospital of Brasilia and the Base Hospital of the Federal District were invited to participate. Inclusion criteria were treatment-naïve patients between 18 and 70 years of age, with mammographic image classification 4C or higher according to Breast Imaging-Reporting and Data System (BI-RADS), and with surgery as primary treatment option. BI-RADS 4C denotes “finding of moderate concern of being cancer” and patients in this category are advised to perform biopsy exams. Exclusion criteria were patients with metastatic or recurrent disease, comorbidity or other disease that prevented the use of fish oil or affected the blood parameters being studied, pacemaker users and those unable to be weighted or with edema. All patients signed an informed consent before entering the study.

### Study design

A randomized, controlled, double-blind study was conducted between the period of February 2012 and March 2013. The study was carried out in compliance with Good Clinical Practice and the Consolidated Standards for Reporting of Trials (CONSORT) statement. The study protocol was approved by the Human Research Ethics Committees of the University of Brasilia and of the Federal District Health Secretariat. The Brazilian Registry of Clinical Trials (ReBEC) is one of the primary registry site of the WHO International Clinical Trials and the study was registered as RBR-2B2hqh. The randomization was done beforehand and performed by manual raffling the blocks of ten sequential numbers with five chances of being raffled to one of the two groups. A laboratory technician not involved in the research performed the randomization, assigned fish oil group (FG) or placebo group (PG) to the sequential numbers and kept the randomized sequence secret to the project team members and patients until the last patient’s data collection were finished. Patients were randomized only after positive biopsy confirmation for malignancy. The same technician provided the blinded supplement (which was identified only with the sequential numbers) to the research team. The supplements were supplied in white plastic bottles containing 30 capsules (sufficient for 15 days). Patients entering the study were assigned to the sequential identification number. The intervention lasted 30 days, immediately following the diagnosis and before the surgical procedure. Thirty days was the mean time needed for patients to go through pre-surgery exams. Patients were scheduled to return in the middle of the intervention period, when the second supplement bottle was given. At the final visit, patients were asked to return any unused capsules.

Twelve hours fasted blood samples were collected for biochemical and immunological analyses at baseline and at the end of the intervention period. For the evaluation of nutritional status, body weight, height and body composition analyses were performed. Dietary intake was evaluated by 24-h recall method, two at baseline and two at the end of intervention.

### Fish oil supplements

Bulk fish oil concentrate (MaxOmega 46/38 EE®, Equateq Ltd., United Kingdom) and mineral oil were purchased and encapsulated (Relthy Laboratories Ltd., Brazil) in 1 g gel capsules. FG patients were asked to ingest 2 g of fish oil concentrate (2 capsules) daily for 30 days, at lunch and dinner times. Each gram of fish oil concentrate contained 470 mg of EPA, 390 mg of DHA plus 18:3n3 acid, in the form of ethyl esters, with a total of 1.81 g of n-3 fatty acids per day, according to the manufacturer’s information and confirmed in our lab. The fish oil capsules also contained 0.32% (*w*/w) of vitamin E (α-tocopherol) as antioxidant. Placebo group patients were given 2 g per day of mineral oil of the same color and smell of the fish oil supplement, divided in 2 capsules of 1 g each. In our study, rather than masking the typical odor of fish oil, the plastic bottles for mineral oil capsules were previously treated with fish oil capsules. This procedure added subtle fish oil smell to the bottles of mineral oil, thus, all patients thought they were receiving fish oil capsules.

Compliance was promoted by regular telephone contact with the patients and was monitored by counting the returned capsules at 15th and 30th day visits. Plasma phospholipid fatty acid profile before and at the end of the intervention was also analyzed for compliance evaluation.

### Nutritional status and dietary intake

Weight and height were measured in a Toledo digital scale and a metal stadiometer attached to the scale, using standard procedure. Body mass index (BMI) was calculated and classified according to the World Health Organization cutoff values [18].

The bioelectrical impedance analysis was performed with BIA Quantum II instrument (RJL Systems®) according to the standardized procedure. The phase angle (PA) was obtained from the arc tangent relationship of reactance/ resistance × 180 / π [19].

Dietary intake was assessed by 24-h recall using the method of multiple passes and nutrient composition was calculated with NutWin (1.5.2.51 version) software. NutWin uses the USDA food composition database for nutrient calculation.

### Blood analysis

Blood samples were obtained for biochemical (serum) and immunological analysis (plasma). Biochemical analysis included serum glucose, total cholesterol, high- and low- density lipoprotein cholesterol and triglycerides (Labtest®), complete blood count (CELL-DYN 3500 system), albumin and high sensitivity C-reactive protein (hsCRP) by immunonephelometry (Siemens®). Immunological parameters evaluated were peripheral blood mononuclear CD4^+^ e CD8^+^ lymphocyte cell counts, plasma cytokines and PGE_2_. Plasma phospholipid fatty acid profile was also analyzed as a marker of compliance.

### Flow-cytometric analysis

The peripheral blood mononuclear cells (PBMC) were obtained by density gradient centrifugation with Histopaque ® - 1077 (Sigma-Aldrich). Lymphocytes were suspended in phosphate buffered saline at a concentration of 5 × 10^5^ cells/ well. CD4^+^ and CD8^+^ cells were counted with surface marker PE mouse anti-human CD4 and CD8 (BD Biosciences, USA). The analysis was performed in a FACSCalibur flow cytometer equipped with CellQuest software (BD Biosciences, USA). Twenty thousand events were acquired from each sample and the results were analyzed using FlowJo software, version 10.0 (Treestar, Inc. USA)*.*

### Proinflammatory cytokines and prostaglandin E_2_

Plasma IL-6, IL-1β and TNF-α cytokines were quantified by the ELISA method (Bioscience, San Diego, USA). Prostaglandin E_2_ metabolites were quantified by competition ELISA method using the Prostaglandin E Metabolite EIA kit (Cayman Chemical Company, USA) according to the manufacturer’s instructions.

### Phospholipid fatty acid profile

Plasma lipid was extracted according to Folch et al. [20] and phospholipids were separated by thin layer chromatography with solvent system hexane: diethyl ether: acetic acid (80:20:2 *v*/v/v) [21]. Phospholipid fatty acids were esterified by acid methylation [21] and analyzed by gas chromatography (GC) (Shimadzu, 17A model), using SP2560 column (Supelco, Bellefonte, PA, USA). Fatty acids were identified using external standards (Sigma®) and the results were expressed as percentage of fatty acid in relation to the total area of the fatty acids.

### Statistical analysis

Primary end points of this study (nutritional status/ body weight) have not been reported in breast cancer patients receiving n-3 fatty acids prior to treatment. The sample size calculation was performed on the basis of Bougnoux et al. study [13], which assessed the effect of DHA in breast cancer patients during chemotherapy (that study found objective response rate to treatment in 44% of patients). Assuming a hypothesis that no more than 5% of placebo group would present a positive response (in immunological or nutritional parameter), we estimated that a minimum sample of 16 subjects in each group would allow the detection of differences due to the effect of n-3 use, with a 80% power and 5% significance level.

Descriptive statistics were presented as percentages, means and standard deviations or median (upper and lower quartiles). Baseline results were analyzed using the chi-square test for categorical variables and Mann Whitney test for continuous variables. To check for intra group differences, the Wilcoxon test was used. Differences between groups were verified by a two-way repeated measures ANOVA for ordinal data with group (fish oil and placebo) as between subject factor and time as within subject factor [22]. All tests were two-tailed and the significance level was set at *p* < 0.05. Analyses were performed using R free software.

## Results

### Study population

**Fig. 1:**
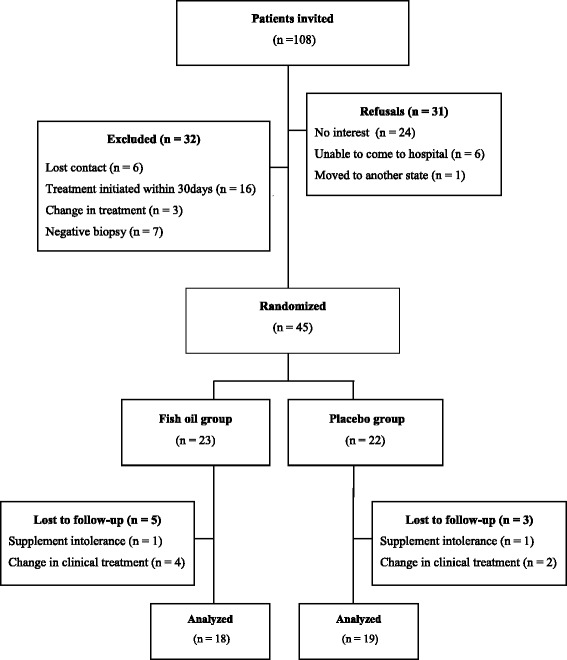
CONSORT flow chart

One hundred and eight patients were invited to participate in the study (Fig. 1). Of these, 77 (71%) accepted the invitation, but 32 patients were excluded due to: loss of contact for the baseline visit (*n* = 6), surgery scheduled to date shorter than 30 days (*n* = 16), change in clinical treatment (*n* = 3) and negative biopsy (*n* = 7). Thus, 45 patients were randomized. Of the randomized patients, eight of them discontinued the study due to supplement intolerance (*n* = 2) and to change to neoadjuvant chemotherapy as primary treatment (n = 6). Thirty seven patients completed the study, of whom 18 were supplemented with fish oil and 19 with placebo.

### Baseline characteristics

The socio-demographic and clinic-pathological characteristics of patients at baseline are shown in Table 1. Most of the patients had infiltrating ductal carcinoma (62%), clinical staging 0/ I/ II (56%), estrogen receptor + (ER+) (72%), progesterone receptor + (PR+) (59%) and negative for human epidermal growth factor receptor 2 (HER2) (56%). There was no significant difference between the FG and PG groups with respect to these variables.

**Table 1:** Socio-demographic and clinicopathological characteristics of patients randomized according to the study group

	Groups		*p* ^a^
	Fish oil (*n* = 18)	Placebo (*n* = 19)	
Age (years) ^b^	48.6 ± 9.0	53.4 ± 7.5	0.107
Education level (years) ^b^	7.2 ± 3.9	9.1 ± 4.0	0.227
Menopause % (n)			
No	50.0 (9)	26.3 (5)	0.138
Yes	50.0 (9)	73.7 (14)	
Histological type % (n)			
Ductal carcinoma in situ (DCIS)	22.2 (4)	10.5 (2)	0.756
Infiltrating ductal carcinoma (IDC)	72.2 (13)	78.9 (15)	
IDC + DCIS	5.6 (1)	5.3 (1)	
No information	–	5.3 (1)	
TNM classification % (n)			
Tumor in situ	16.7 (3)	–	–
I	16.7 (3)	5.3 (1)	0.212
II	38.9 (7)	47.4 (9)	
III	27.8 (5)	42.1 (8)	
No information	–	5.3 (1)	
Estrogen receptor (ER) % (n)			
ER+	77.8 (14)	73.7 (14)	1.000
ER-	16.7 (3)	15.8 (3)	
No information	5.6 (1)	10.8 (2)	
Progesterone receptor (PR) % (n)			
PR+	66.7 (12)	57.9 (11)	1.000
PR-	27.8 (5)	31.6 (6)	
No information	5.6 (1)	10.5 (2)	
Human epidermal growth factor receptor 2 (HER2) % (n)			
HER2+	33.3 (6)	26.3 (5)	1.000
HER2-	61.1 (11)	63.2 (12)	
No information	5.6 (1)	10.5 (2)	

*TNM* Tumor, node, metastasis

^a^ Mann-Whitney test for age and education level and chi-square for the other variables

^b^ Mean ± standard deviation

According to the BMI classification, the majority of the patients had excess weight, 43% being classified as overweight and 30% as obese. No between group differences existed in the anthropometric parameters and intake variables. The daily consumption of EPA and DHA was low in both groups, with medians of 0.005 g/ day of EPA and 0.020 g/ day of DHA, among FG patients and 0.005 g/day and 0.025 g/day, respectively, in the PG.

The percentage of baseline plasma phospholipid EPA were 0.4% and 0.3% in FG and PG, respectively; while DHA were 2.5% and 3.1%, with no group differences. The FG had significantly lower percentage of oleic acid (*p* = 0.027) and a higher ratio of 18.0/18.1 (*p* = 0.022) when compared with PG. The percentage of other fatty acids was similar between the groups.

There was no significant difference between the groups regarding the baseline percentage and ratio of PBMN CD4^+^ and CD8^+^ lymphocytes, serum levels of proinflammatory cytokines (TNF-α, IL-6 and IL-1β), PGE metabolites and hsCRP. With the exception of monocytes, blood count and serum biochemical parameters were similar between FG and PG.

### Tolerability and compliance

Among the patients who completed the study, 55% and 47% of the FG and PG patients, respectively, reported side effects such as dizziness, nausea, frequent belching, increased bowel frequency, heartburn and gastric fullness. However, no between group differences was observed for the presence of symptoms (*p* = 0.616). Despite the reported side effects, 92% and 93% of the prescribed capsules were consumed in the FG and PG, respectively, which was considered as good supplement compliance.

### Intervention effects

The effects of the intervention on nutritional status and dietary intake are shown in Table 2. At the end of the intervention period, the FG patients presented significant gain of fat mass (*p* = 0.029), but no difference was observed between the groups regarding this and other anthropometric parameters analyzed. There was no intra group difference in the macronutrient intake, both in the PG and FG patients. However, there was a between group difference in energy (*p* = 0.038) and protein (*p* = 0.010) ingestion being higher in PG. The FG group intake of monounsaturated, palmitic, stearic and oleic fatty acids reduced significantly, however, with no between group differences. The dietary EPA, DHA and total n-3 fatty acids showed no intra or between group differences at the end of intervention period (Table 2).

**Table 2:** Nutritional status and dietary intake at baseline and at the end of the study

	Fish oil group (n = 18)				*P* ^*a*^	Placebo group (n = 19)				*P* ^*a*^	*P* ^b^
	Initial		Final			Initial		Final			
	Median	IQR	Median	IQR		Median	IQR	Median	IQR		
Nutritional status											
Weight (kg)	67.3	62.0–74.1	67.5	62.8–77.6	0.078	66.6	57.7–73.2	67.5	57.5–71.7	0.776	0.079
BMI (kg/m^2^)	27.0	23.7–32.0	27.1	23.6–32.5	0.078	26.6	24.9–30.2	26.3	24.8–29.6	0.723	0.101
Lean body mass (kg)	41.5	39.6–45.7	41.0	45.0–43.2	0.170	40.5	34.8–44.0	40.4	34.8–43.8	0.660	0.406
Fat mass (kg)	26.3	19.5–33.0	26.8	21.9–34.6	0.029	26.5	21.6–30.2	24.3	22.1–29.8	0.977	0.101
% Body fat	37.7	28.1–44.9	38.1	33.2–46.0	0.149	38.9	37.0–43.8	39.4	35.9–42.0	0.820	0.298
SPA	−0.6	−1.2 - -0.2	−0.7	−1.1 - 0.1	0.513	−1.2	−1.6 - -0.6	−1.1	−1.63 - -0.7	0.394	0.492
Dietary intake											
Energy (kcal)	1451	1052–1755	1226	1011–1629	0.124	1162	991–1500	1289	1186–1480	0.520	0.038
Kcal/kg	21	16–27	17	14–24	0.173	20	14–22	20	16–23	0.877	0.259
Carbohydrates (g)	172	128–260	155	118–235	0.148	147	133–186	171	118–226	0.557	0.200
Protein (g)	64	48–80	47	42–60	0.124	51	44–68	62	47–81	0.184	0.010
Lipids (g)	45	34–66	42	37–51	0.124	42	33–53	38	27–53	0.546	0.686
*Fat acids (g)*											
*Saturated*	11.1	7.6–15.7	9.4	8.1–13.5	0.163	8.9	7.5–13.4	9.5	6.7–13.5	0.936	0.536
*Monounsaturated*	11.6	9.1–19.3	10.9	7.5–13.7	0.039	10.5	8.6–14.7	9.4	6.9–15.0	0.673	0.747
*Polyunsaturated*	9.4	7.4–11.5	8.1	7.2–10.6	0.163	9.0	7.1–10.6	8.0	6.5–10.0	0.376	0.752
*16:0*	6.3	4.6–9.7	5.3	3.9–7.0	0.013	5.3	4.5–7.6	5.2	3.9–7.5	0.809	0.449
*18;0*	2.6	1.9–4.6	2.4	1.5–3.4	0.019	2.1	1.8–3.3	2.2	1.6–3.8	1.000	0.489
*18:1n-9*	10.7	8.4–17.8	10.1	7.0–12.7	0.049	9.5	7.7–13.6	8.5	6.3–14.6	0.629	0.838
*18:2 n-6*	7.8	6.9–9.9	7.0	6.3–9.7	0.177	7.9	6.1–9.3	7.0	5.6–8.5	0.243	0.842
*18:3 n-3*	0.9	0.7–1.1	0.8	0.7–1.1	0.981	0.8	0.6–0.8	0.8	0.5–0.9	0.794	0.776
*20:4n-6*	0.10	0.47–0.17	0.07	0.54–0.11	0.163	0.09	0.05–0.12	0.09	0.06–0.19	0.162	0.165
*20:5n-3 (EPA)*	0.005	0.000–0.007	0.005	0.000–0.010	0.633	0.005	0.000–0.010	0.005	0.000–0.015	0.395	0.334
*22:6n-3 (DHA)*	0.020	0.002–0.052	0.020	0.005–0.032	0.162	0.025	0.010–0.030	0.015	0.000–0.065	0.139	0.295
*Total n-3*	1.0	0.7–1.3	0.8	0.8–11	0.850	0.8	0.6–1.1	0.8	0.6–1.0	0.831	0.711
*Total n-6*	8.3	7.1–10.2	7.2	6.4–9.7	0.201	8.0	6.2–9.4	7.0	5.2–8.4	0.163	0.850
*n-6/n-3 ratio*	8.3	5.8–9.7	7.8	7.4–8.8	0.723	8.3	7.3–10.1	7.2	6.7–10.5	0.381	0.175
*18:0/18:1 ratio*	0.2	0.2–0.2	0.2	0.2–0.2	0.554	0.2	0.2–0.2	0.2	0.2–0.2	0.469	0.387

*IQR* Interquartile range, *BMI* Body mass index, *SPA* Standardized phase angle, *EPA* Eicosapentaenoic acid, *DHA* Docosahexaenoic acid

^a^ Intragroup differences according to Wilcoxon test

^b^Interaction test of a two-way repeated measures ANOVA for ordinal data to verify the significance of differences between fish oil and mineral oil groups

Significant increase in plasma total n-3 fatty acids (*p* = 0.004) and decrease in n-6: n-3 ratio (*p* = 0.002) was seen in FG patients, with a significant between group differences (*p* = 0.005 and *p* = 0.012, respectively) (Table 3).

**Table 3:** Blood fatty acids profile at baseline and at the end of the study in both groups

	Fish oil group (*n* = 18)				*P* ^*a*^	Placebo group (*n* = 19)				*P* ^*a*^	*P* ^b^
	Initial		Final			Initial		Final			
	Median	IQR	Median	IQR		Median	IQR	Median	IQR		
Fatty acids											
*Saturated*	58.7	50.4–63.5	58.4	50.6–64.4	0.863	52.2	49.1–60.7	55.2	48.9–59.1	0.601	0.857
*Monounsaturated*	10.1	9.0–11.3	9.8	8.5–10.9	0.130	9.8	9.2–12.2	10.6	8.5–11.9	0.809	0.326
*Polyunsaturated*	27.4	21.9–33.3	27.9	25.0–38.0	0.113	36.1	25.4–37.7	35.2	28.2–38.8	0.376	0.795
16:0	29.9	24.6–35.1	30.4	23.6–34.7	0.356	25.0	22.1–31.3	25.7	22.7–29.6	0.717	0.824
18:0	16.8	15.3–17.4	17.4	15.7–18.6	0.356	15.3	13.7–18.5	15.9	12.9–17.1	0.841	0.409
18:1n-9	4.3	3.5–5.2	4.4	3.7–5.8	0.943	5.2	4.5–6.5	5.1	4.4–6.5	0.629	0.525
20:4n-6	8.8	7.3–10.9	8.2	6.0–10.2	0.124	10.0	7.9–14.0	11.1	8.5–12.6	0.984	0.284
20:5n-3 (EPA)	0.4	0.1–0.8	1.5	0.9–2.1	0.004	0.3	0.0–0.8	0.5	0.0–1.2	0.293	0.034
22:6n-3 (DHA)	2.5	1.9–3.6	4.6	3.4–6.2	0.007	3.1	2.1–5.0	3.8	2.0–4.9	0.904	0.000
Total n-3	3.3	2.4–4.9	6.5	4.3–8.7	0.004	3.7	2.6–5.9	4.1	2.9–5.9	0.952	0.005
Total n-6	25.0	19.1–30.2	23.0	19.1–29.5	0.554	31.6	22.6–32.4	30.0	24.1–33.9	0.702	0.246
n-6:n-3 ratio	7.7	5.3–9.7	3.8	3.0–4.7	0.002	7.0	4.5–11.1	6.8	4.4–8.8	0.904	0.012

*IQR* Interquartile range, *EPA* Eicosapentaenoic acid, *DHA* Docosahexaenoic acid

^a^Intragroup differences according to Wilcoxon test

^b^Interaction test of a two-way repeated measures ANOVA for ordinal data to verify the significance of differences between fish oil and mineral oil groups

**Fig. 2:**
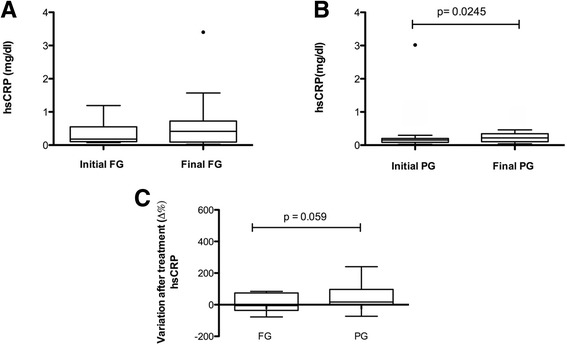
Changes in high sensitivity C-reactive protein (hsCRP) according to the study groups. **a** Fish oil group (FG), *n* = 15 (**b**) Placebo group (PG), *n* = 16; *p*-value for the Wilcoxon test (**c**) Variation after treatment (Δ%) n = 15; *p* values for Mann Whitney test. Data are presented as medians, upper and lower quartiles, maximum and minimum values. (•) Outlier values indicated in the chart were excluded from the statistical analyses

Regarding the acute phase immunological response, no significant change was observed in the FG (initial median 0.1 [IQR 0.1–0.5], final median 0.3 [IQR 0.0–0.7], *p* = 0.510) while in PG patients there was a significant increase in hsCRP (initial median 0.1 [IQR 0.0–0.2], final median 0.2 [IQR 0.1–0.3], *p* = 0.024). While hsCRP remained stable in patients supplemented with n-3 fatty acids, the PG patients had a more pronounced increase in serum hsCRP levels, with a non-significant between group difference (FG Δ% = −5.9 [−35.4–74.12], PG Δ% = 17.2 [−0.16–91.99] *p* = 0.059) (Fig. 2). No significant changes in serum TNF-α, IL-1β, IL-6 cytokines were observed.

**Fig. 3:**
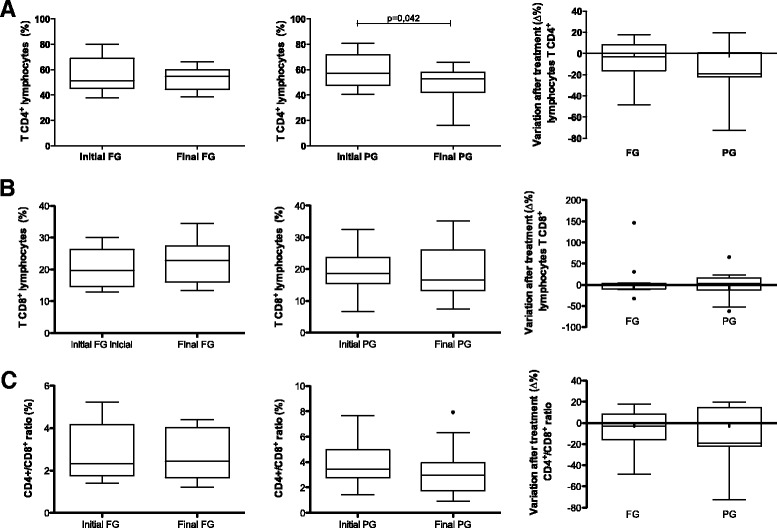
Variation after treatment (Δ%) of subpopulation of CD4^+^ T lymphocytes, CD8^+^ and CD4^+^/CD8^+^ ratio, according to the study groups. Fish oil group (FG); Placebo group (PG); (**a**) CD4^+^ T lymphocytes, PG *n* = 12, FG n = 15; p-value for the Wilcoxon test (**b**) CD8^+^ T Lymphocytes, PG *n* = 14, FG *n* = 13 (**c**) CD4^+^/CD8^+^ ratio, PG n = 12 and FG *n* = 15. Data are presented as medians, upper and lower quartiles, maximum and minimum values

We observed a significant reduction in the percentage of CD4^+^ T lymphocytes in the peripheral blood of PG patients (initial median 57.2 [IQR 47.7–71.8], final median 52.7 [IQR 42.3–57.9], *p* = 0.042) and no change in the percentage of CD8^+^ cells. In the FG, no change in the percentages of CD4^+^ and CD8^+^ T cells and CD4^+^/ CD8^+^ ratio occurred. No between group effects of treatment (Δ%) were observed for these parameters (Fig. 3). Serum PGE metabolite levels in both groups did not change due to intervention. Serum glucose, total cholesterol and fractions, complete blood count and serum albumin showed no within or between group differences (Table 4).

**Table 4:** Biochemical parameters at baseline and at the end of the study in both groups

	Fish oil group (n = 18)				*P* ^*a*^	Placebo group (n = 19)				*P* ^*a*^	*P* ^b^
	Initial		Final			Initial		Final			
	Median	IQR	Median	IQR		Median	IQR	Median	IQR		
Biochemical											
RBC (×10^6^/mm^3^)	4.7	4.5–5.1	4.7	4.5–4.9	0.254	4.7	4.4–5.2	4.7	4.4–4.9	0.493	0.941
Hemoglobin (g/dL)	14.0	12.8–15.2	13.7	13.0–14.70	0.069	14.0	13.1–14.9	13.8	13.0–14.4	0.623	0.426
Hematocrit (%)	41.7	38.3–45.7	41.1	39.1–43.6	0.139	42.2	38.6–44.0	41.6	39.0–43.6	0.877	0.785
Leucocytes (mm^3^)	6405	5342–7950	6910	5105–7750	0.795	5220	4150–6700	5780	5265–6590	0.653	0.521
Platelets (×10^3^/mm^3^)	281.0	215.2–307.7	259.0	220.0–291.0	0.523	246.0	193.0–282.0	241.0	212.0–278.5	0.492	0.456
Albumin (g/dL)	4.4	4.1–4.5	4.2	4.1–4.4	0.153	4.3	4.2–4.5	4.3	4.0–4.4	0.319	0.818
Fasting Glucose (ml/dL)	95.0	87.0–103.2	91.0	83.2–101.5	0.351	90.0	84.0–98.0	92.5	84.7–98.2	0.410	0.126
Total cholesterol (mg/dL)	218.0	194.5–258.5	217.0	194.5–254.5	0.758	211.0	196.0–247.0	208.0	183.5–241.5	0.185	0.414
HDL (mg/dL)	45.0	39.5–50.2	44.0	38.0–48.5	0.476	46.0	41.0–50.0	47.0	40.5–54.7	0.905	0.689
LDL (mg/dL)	141.0	118.7–176.7	146.0	122.0–176.0	0.518	145.0	121.0–169.0	140.5	112.0–157.2	0.138	0.182
Triglycerides (mg/dL)	160.0	90.7–199.5	146.0	98.0 - 191.5	0.421	125.0	75.0–165.0	105.0	80.0–146.7	0.679	0.887

*IQR* Interquartile range, *RBC* Reed blood cells, *HDL* High-density lipoprotein, *LDL* Low-density lipoprotein

^a^Intragroup differences according to Wilcoxon test

^b^Interaction test of a two-way repeated measures ANOVA for ordinal data to verify the significance of differences between fish oil and mineral oil groups

## Discussion

To our knowledge, this randomized controlled double blind trial is the first that investigated the effects of supplementation with n-3 fatty acids in newly diagnosed breast cancer patients, prior to treatment. In the study, the FG plasma EPA and DHA levels increased significantly after 30 days of n-3 supplementation. In terms of immune parameters, whereas hsCRP significantly increased and CD4^+^ reduced in the placebo group, in the n-3 fatty acids suplemmented patients serum hsCRP and CD4^+^ were kept at levels similar to baseline values.

CRP is an acute-phase serum protein of the pentraxin family produced mainly by hepatocytes and is regulated at the transcriptional level by IL-6. Its plasma concentration increases during inflammatory state [23]. In our study, the placebo group showed an increase in CRP levels, suggestive of an inflammatory response to the tumor, while, in n-3 fatty acids treated-breast cancer patients, the CRP showed a more regulated response. We speculate that n-3 fatty acid suplementation might have modulated the inflammatory response to tumor, which in turn could collaborate to a better evolution of the patient during subsequent treatment period. The absence of similar increase in IL-6 in our study may relate to the differences in the kinetic of their production, in which IL-6 serum levels had already decreased while CRP was still increasing, when tested in the study [23].

These results are consistent with the idea of EPA and DHA acting in the modulation of CRP dependent inflammatory responses. Similar results have been observed in patients with advanced cancer [17, 24]. These results are relevant, given that high levels of CRP have been previously associated with a worse prognosis in breast cancer patients [10] and with the fact that the results could potentially be attributed to n-3 fatty acids supplementation. Our results are also consistent with the potential preventive effect of n-3 fatty acids in breast cancer [12].

According to Calder [25], dietary n-3 fatty acids should be incorporated into leukocyte membrane in order to be an effective immunomodulator. In breast cancer patients, after oral supplementation with 3 g of polyunsaturated fatty acids n-3 (EPA and DHA) there was a threefold increase in circulating total n-3 acids [26]. In the present study, plasma phospholipd fatty acids were used as surrogate markers of compliance to the n-3 intervention and after 30 days, the median increase was significant but inferior to those reported by Bagga et al. [26]. These differences in incorporation may relate to the amount of n-3 fatty acids supplemented in our study (1.8 g/ day) that could have been insufficient for a higher incorporation. Of note, recent study has indicated that different lipid structures used for EPA and DHA supplementation have similar rates of incorporation into the blood [27].

Low peripheral blood CD4^+^ counts [5, 6] have been observed even in the early stages of breast cancer patients. Whereas the number of circulating T CD4+ lymphocytes decreased in the placebo group, which is in line with the suppressor substances produced by tumor cells as its immune escape mechanisms, the maintenance of the number of T CD4+ lymphocytes in the n-3 fatty acid treated group may have been due to the proliferative effect of fatty acids on lymphocyte functions [2]. In patients of the placebo group, although the number of TCD8^+^ lymphocyte did not change, the possibility that the lower number of TCD4^+^ lymphocytes might have impaired proliferative capacity of the TCD8^+^ cells cannot be ruled out, because helper function of TCD4^+^ lymphocytes is required to full activation of TCD8^+^ cells [28]. As the number of TCD4^+^ and TCD8^+^ lymphocytes and its ratio remained stable in the fish oil treated group, taken together, the results of our study could suggest a positive effect of fish oil supplement in the adaptive immunity. Surgery is the mainstay of treatment of these patients and this procedure induces substantial immunomodulation, with pro-inflammatory response and leukocytosis [29]. Thus, a balanced adaptive immune response may help prevent postsurgery immunosupression and risks such as tumor dissemination into the circulation [30].

No significant changes were observed in serum proinflammatory cytokines due to the intervention. Similar results in patients with different types of cancer and antineoplastic treatments were reported [14, 31]. Faber et al. [14] supplemented radiotherapy cancer patients with 3.6 g of n-3 fatty acids for 7 days and changes in the serum proinflamatory cytokines were undetectable to some and not significant to IL-6 and IL-8. Moreover, unlike the results of the present study, they observed a reduction in serum PGE_2_ levels. Gomez-Candela et al. [31] did not observe reduction of proinflammatory cytokines, but a tendency of increased serum IL-6 after supplementation with EPA and DHA. Nevertheless, it should be considered that cytokines are mainly produced at local levels, so that one can not exclude the possibility that there were modifications in their local levels but that they were not sufficient to modify the systemic serum levels. We were unnable to find previous studies reporting the effects of n-3 supplementation on circulating cytokines of breast cancer patients.

Despite the plausibility of antineoplastic effect of n-3 fatty acids according to cell culture and animal studies, reports of clinical trials are scarce [32] and the results are inconsistent, one of the reasons being the high variability in the study design. To our knowledge, in the few studies with breast cancer patients, fish oil was studied only as adjuvant to chemotherapy [13, 15, 33]. In our study, the lack of significant findings in relation to proinflammatory cytokines and PGE_2_ may be in part due to the amount of supplement used or the length of the intervention, that could have been insufficient to be effective. Our intervention have used n-3 dose similar to that used by Bougnoux et al. [13], who reported good tolerance and no side effects. However, according to Mocelim et al. [34], when supplementation is carried out during a short period, higher doses of n-3 fatty acids are required to have an antiinflammatory effect. Also, the use of α-tocopherol as antioxidants in fish oil capsules may have reduced the effect of n-3 fatty acids, as demonstrated in experimental studies [35]. Other limitation of the study pertains to the discrepancy between the number of invited patients (*n* = 108) and the patients examined (*n* = 37) which affected the study power. Carrying out the study with patients immediately after the diagnosis of such severe disease was challenging for both the research group and patients, and contributed to high refusal and drop out rates. A positive feature of the study was the good compliance to fish oil supplement (92%), similar to the study by Taylor et al. [24]. As well, the use of mineral oil as placebo had the merit of avoiding the confounding effect of n-6 fatty acids in the control group. As study participants were treatment naïve, the results may better reflect the patient’s metabolic response to the effect of n-3 fatty acids.

## Conclusions

In conclusion, the supplementation of newly diagnosed breast cancer patients with 1.8 g of EPA and DHA for 30 days led to a significant change in the composition of plasma fatty acids, maintained the level of CD4^+^ T cells and serum levels of CRP, suggestive of a beneficial effect on the immune system. Studies considering the molecular subtypes and clinical staging of the disease would further confirm the results presented.

## Abbreviations

BI-RADS: Breast Imaging-Reporting and Data System
BMI: Body mass index
CRP: C-reactive protein
DHA: Docosahexaenoic acid
EPA: Eicosapentaenoic acid
ER+: Estrogen receptor+
FG: Fish oil group
HER2: Human epidermal growth factor receptor 2
hsCRP: High sensitivity C-reactive protein
IL-1β: Interleukin-1β
IL-6: Interleukin-6
IR: Immune response
PA: Phase angle
PBMC: Peripheral blood mononuclear cells
PG: Placebo group
PGE_2_: Prostaglandin E_2_
PR+: Progesterone receptor+
TNF-α: Tumour necrosis factor alpha
